# Editorial: Neovascularization, Angiogenesis and Vasculogenic Mimicry in Cancer

**DOI:** 10.3389/fonc.2020.01140

**Published:** 2020-07-16

**Authors:** César López-Camarillo, Erika Ruiz-García, Naureen Starling, Laurence A. Marchat

**Affiliations:** ^1^Posgrado en Ciencias Genómicas, Universidad Autónoma de la Ciudad de México, Mexico City, México; ^2^Laboratorio de Medicina Translacional y Departamento de Tumores Gastro-Intestinales, Instituto Nacional de Cancerología, Mexico City, México; ^3^Royal Marsden NHS Foundation Trust London, London, United Kingdom; ^4^Programa en Biomedicina Molecular y Red de Biotecnología, ENMyH-Instituto Politécnico Nacional, Mexico City, México

**Keywords:** vasculogenic mimicry, angiogenesis, lncRNAs, microRNAs, therapy

Vasculogenesis refers to the development of new vessels from primordial endothelial stem cells, whereas angiogenesis denotes the formation of vessels from pre-existing capillary structures. In particular, angiogenesis is a complex cellular mechanism required for the formation of new blood vessels from the existing vasculature or from bone marrow-derived endothelial progenitors, allowing homeostasis. In contrast, pathological angiogenesis found in tumors leads to accelerated tumor growth and development at early stages of carcinogenesis (1). During angiogenesis a plethora of pro-angiogenic factors are secreted by cells present in tumor microenvironment including cancer cells, cancer associated-fibroblasts, pericytes, and immune cells to induce vascularization via activation of pre-existing host endothelium. On the other hand, vasculogenic mimicry (VM) describe the ability of tumors cells to organize themselves in patterned three-dimensional (3D) channel-like structures resembling vascular networks in order to acquire nutrients and supports the requirements for tumor growth. VM has been reported in diverse types of tumors such as breast, melanoma, lung, glioblastoma, ovarian, and prostate cancers, among others, indicating that it may be designated as a novel cancer hallmark (2). Importantly, the existence of tumoral VM in oncologic patients has been associated with low free-disease survival and worst prognosis. VM operates in an independent via, or simultaneously, with classical endothelial vessels and angiogenesis. These cellular processes are regulated by diverse cellular lineages including pericytes and multiple pro-angiogenic and anti-angiogenic factors activated by hypoxic microenvironment, which become deregulated in tumors leading to pathological angiogenesis. Pericytes secrete growth factors that stimulate endothelial cells proliferation, and migration; as well as proteases secretion that contribute to modulate the surrounding extracellular matrix (3). In addition, pericytes are recruited by VM-positive cells in order to stimulate sprouting and to provide structural support of the growing vascular-like networks (4). Also, multiple proteins, signaling networks and regulatory non-coding RNAs (ncRNAs) activate cell proliferation, extracellular matrix remodeling, invasion and metastasis mechanisms to promote neovascularization, angiogenesis and VM. However, the balance and interplay among signaling transductors, and ncRNAs is poorly understood. Therefore, there is a great interest in the development of anti-angiogenesis and anti-vasculogenic mimicry strategies that could inhibit tumor vascularization.

In this Research Topic we have organized a collection of original research and review articles that examine the more recent progress in neovascularization, angiogenesis, and VM. To incite readers to check the complete collection, here we introduce several representative contributions made for authors. After the first study published by Maniotis et al. (5), the presence of VM and the experimental approaches used to confirm its presence in tumors and cell cultures have remained controversial. In an opinion paper on VM, Valdivia et al., describe the state of art, and the contentious topics around VM. Authors debate about the utility of the current tools used to demonstrate the presence of VM, specifically the Periodic Acid Schiff (PAS) positive stain of tumor tissues and the commonly used *in vitro* models. They conclude that intercellular connections occurring in cell monolayers when cultured in a Matrigel matrix at early times could not represent VM. Moreover, authors raise doubts on the validity of PAS+ staining to detect the presence of VM in patient tissues, and finally outline the requirement for new biomarkers of VM for clinical use. Remarkably, authors make an urgent call for reliable *in vitro* and *in vivo* VM models which must be approved by the scientific community in order to better explain the mechanisms governing this phenomenon.

Two reviews summarize the actual state of the art in the molecular mechanisms of VM in breast and ovarian cancers. Andonegui-Elguera et al. summarize the mechanisms of VM development in breast cancer, including the participation of signaling proteins and the functional relationships between cancer stem cells, the epithelial-mesenchymal transition and VM. Also, they discuss the clinical significance of VM in prognosis with special emphasis in the opportunities of targeting VM for triple negative breast cancer therapy. Ayala-Domínguez et al. summarize the mechanisms of VM in gynecological ovarian cancer. They reviewed the actual knowledge of angiogenesis, vasculogenesis, and vessel co-option mechanisms with a special focus in the signaling pathways and microRNAs involved in VM regulation. In addition, authors discuss the clinical implications of the potential targeting of molecules involved in VM for ovarian cancer therapy.

Tumor hypoxia is one of the most important mechanisms to activate angiogenesis and VM (6). Salinas-Vera et al. examine the role of hypoxia-regulated microRNAs (dubbed as hypoxamiRs) during the onset of VM in ovarian cancer. They identify the modulation of 11 hypoxamiRs which are predicted to participate in VM and angiogenesis with potential clinical implications. Also, authors demonstrate that miR-765 modulates the initiation of 3D capillary-like arrangements via activation of the VEGFA/AKT1/SRC axis in SKOV3 ovarian cancer cells.

The limited clinical outcome of anti-angiogenic therapy depends, at least in part, on the inefficient tumor perfusion that limits both the diffusion of chemotherapeutic agents and the antitumoral functions of immune cells. Pathological tumor angiogenesis is characterized by an immature and disorganized vasculature architecture leading to enhanced permeability and retention effect which may results in cancer cells intravasation and increased metastasis (7). Therefore, vascular normalization has emerged as a new concept and a complementary therapeutic approach aiming to normalize the tumor vasculature. Mattheolabakis and Mikelis describe an overview of the nanoparticles used for simultaneous delivery of anti-angiogenic and chemotherapeutic drugs, which may take advantage of the leaky and tortuous tumor vasculature to diffuse out of the tumor vessels, aiming to achieve vascular normalization and higher efficacy for anti-cancer therapies.

In a mini review paper, Fernández-Cortés et al. discuss the role of tumor microenvironment constituted by tumor associated macrophages, cancer-associated fibroblasts, cancer stem cells, stromal cells and pericytes in VM acquisition. They emphasize on the phosphorylation of the VE-cadherin frequently expressed in endothelial cells and diverse types of aggressive tumors, and its role in VM formation. Also, authors describe the current therapeutic agents targeting FAK/Y658 VE-cadherin and VE-PTP/TIE-2 which have been proposed to impair VM. Another study by Delgado-Bellido et al., showed that melanoma cells undergoing VM express the VE-cadherin phosphatase VE-PTP which complexed with VE-Cadherin and p120. VE-PTP knockdown results in degradation of complex and enhanced autophagy suggesting a pivotal role for VE-PTP in VM formation.

In the last decade, the altered expression of ncRNAs such as microRNAs and long non-coding RNAs (lncRNAs) have been reported in diverse types of tumors where they regulate the expression of oncogenes and tumor suppressor genes driving tumorigenesis. Four reviews focus on the pivotal roles of ncRNAs in cancer are presented in this Research Topic. López-Urrutia et al., review the crosstalk between lncRNAs, microRNAs and mRNAs with a special emphasis in neovascularization, VM, and angiogenesis. Authors consider that the current knowledge on the lncRNA/microRNAs/mRNA axis in these related cellular processes is still limited and deserves further scrutiny. Likewise, Hernández de la Cruz et al., discuss the role of tumor microenvironment, the epithelial-mesenchymal transition and signaling pathways in VM formation in solid tumors. Also, they describe the regulation networks of lncRNAs-microRNAs and their potential impact in personalized cancer treatments. Authors remark that microRNAs and lncRNAs can be potential biomarkers for prognosis, and predictors of therapy response. An epigenetic perspective on the regulatory roles of ncRNAs in neovascularization and angiogenesis in normal and cancer cells is provided by Hernández-Romero et al.. Authors describe several microRNAs and their epigenetic targets involved in angiogenesis and vascular diseases. Also the role of lncRNAs as scaffolds for epigenetic players in neovascularization and angiogenesis is discussed. They conclude that microRNAs and lncRNAs could influence the epigenetic mechanisms in endothelial cells and tumors, thus making ncRNAs as promising epigenetic targets for therapy. Finally, Cao et al. describe the role of ncRNAs regulating the mechanisms of lymphangiogenesis in lymphatic development and discuss their potential as therapeutic targets.

Gastric and esophageal cancers are the third and sixth leading causes of cancer related death worldwide, respectively. Butters et al. present a review about the progress in targeting VGFA and the immunotherapy combination strategies in oesophagogastric cancer. Authors summarize the phase III studies targeting VEGF and the clinical trials focused in the study of immune-checkpoint inhibitors and anti-angiogenic compounds in OG cancer therapy. On the other hand, Lizárraga-Verdugo et al., review the actual knowledge of CSCs research in gastrointestinal cancers (GIC). Cancer stem cells (CSCs) are a small subpopulation of cells present in discrete tumor niches that exhibit a stem-cell phenotype similar to progenitors such as self-renewal, differentiation and maintenance of tumor growth and heterogeneity. Such cells have been found and isolated from diverse types of tumors, and they represent attractive targets for therapy (8). Authors remark that CSCs from GIC are able to transdifferentiate into endothelial-like cells and pericytes, two important lineages for maintenance of cancer vascular niche, thus opening opportunities for therapy intervention of angiogenesis and VM.

Quintero-Fabián et al. emphasize the importance of the matrix metalloproteinases (MMPs) that participate in the degradation of basement membrane to stimulates the cancer cell growth and spreading in various types of cancer. They also analyze the roles of MMPs, cytokines, and immune system cells in the angiogenic events in cancer cells.

In conclusion, investigations of vascular diseases continue to be essential toward the development of new therapeutic strategies that produce more successful treatments for localized and metastatic cancers. We believe that the experimental discoveries and opinions presented in this Research Topic may have a major impact on oncology research and treatment and will inspire future research. We hope this Research Topic will fuel further interests in Scientifics, general readers and Scholars.

## Author Contributions

All authors listed have made substantial contributions to the Research Topic, and approved it for publication.

## Conflict of Interest

The authors declare that the research was conducted in the absence of any commercial or financial relationships that could be construed as a potential conflict of interest.
